# Image Quality of 3^rd^ Generation Spiral Cranial Dual-Source CT in Combination with an Advanced Model Iterative Reconstruction Technique: A Prospective Intra-Individual Comparison Study to Standard Sequential Cranial CT Using Identical Radiation Dose

**DOI:** 10.1371/journal.pone.0136054

**Published:** 2015-08-19

**Authors:** Holger Wenz, Máté E. Maros, Mathias Meyer, Alex Förster, Holger Haubenreisser, Stefan Kurth, Stefan O. Schoenberg, Thomas Flohr, Christianne Leidecker, Christoph Groden, Johann Scharf, Thomas Henzler

**Affiliations:** 1 Department of Neuroradiology, University Medical Center Mannheim, Medical Faculty Mannheim, Heidelberg University, Mannheim, Germany; 2 Institute of Clinical Radiology and Nuclear Medicine, University Medical Center Mannheim, Medical Faculty Mannheim, Heidelberg University, Mannheim, Germany; 3 Siemens Healthcare Sector, Division of Computed Tomography, Forchheim, Germany; University of Nebraska Medical Center, UNITED STATES

## Abstract

**Objectives:**

To prospectively intra-individually compare image quality of a 3^rd^ generation Dual-Source-CT (DSCT) spiral cranial CT (cCT) to a sequential 4-slice Multi-Slice-CT (MSCT) while maintaining identical intra-individual radiation dose levels.

**Methods:**

35 patients, who had a non-contrast enhanced sequential cCT examination on a 4-slice MDCT within the past 12 months, underwent a spiral cCT scan on a 3^rd^ generation DSCT. CTDI_vol_ identical to initial 4-slice MDCT was applied. Data was reconstructed using filtered backward projection (FBP) and 3^rd^-generation iterative reconstruction (IR) algorithm at 5 different IR strength levels. Two neuroradiologists independently evaluated subjective image quality using a 4-point Likert-scale and objective image quality was assessed in white matter and nucleus caudatus with signal-to-noise ratios (SNR) being subsequently calculated.

**Results:**

Subjective image quality of all spiral cCT datasets was rated significantly higher compared to the 4-slice MDCT sequential acquisitions (p<0.05). Mean SNR was significantly higher in all spiral compared to sequential cCT datasets with mean SNR improvement of 61.65% (p*_Bonferroni0.05_<0.0024). Subjective image quality improved with increasing IR levels.

**Conclusion:**

Combination of 3^rd^-generation DSCT spiral cCT with an advanced model IR technique significantly improves subjective and objective image quality compared to a standard sequential cCT acquisition acquired at identical dose levels.

## Introduction

Cranial computed tomography (cCT) is the first-line imaging modality of choice in cases of trauma and other acute neurological emergencies. For none-enhanced cCT imaging, there are two competing techniques: the sequential (incremental) and the spiral (helical) CT.

Since its invention by Kalender in the 1980s [1, 2], spiral (helical) CT has developed into a firmly established method especially employed in whole body imaging [3]. In particular since the introduction of multi-detector row technology, the major advantage is the short acquisition time with the possibility of large volume scanning [1]. Moreover, spiral CT acquisitions enables calculating overlapping images, which improves 2- and 3- dimensional secondary reconstructions [4]. However, since the introduction of CT in 1972, routine CT scans of the brain have usually been made with a sequential technique. Low acceptance for spiral cCT is mainly due to the traditionally presumption of superior image quality using the sequential mode in the field of neuroradiology [5, 6]. Some authors hold the view that delineation of structures with low contrast differences like grey and white matter in spiral acquisitions is inferior to a sequential acquisition technique and beam hardening artifacts localized close to the skull are more pronounced potentially mimicking intracranial hemorrhage [5]. Other authors argue, that there might be no demand for rapid scanning as brain and spine imaging lacks of motion artifacts due to respiration [6]. Nonetheless, especially polytraumatized patients and patients with acute neurological disorders such as cerebral ischemia or intracranial hemorrhage might benefit from a short acquisition time using spiral acquisitions, as they are agitated and often difficult to handle during the CT examination [7]. Significantly, these cCTs have an immediate impact on therapy: Artifacts localized close to the skull in spiral cCT in acute brain infarction frequently resemble bleeding, which prohibits the crucial use of consecutive thrombolysis therapy. Consequently, there is an exceeding demand for cCT which unites high quality of conventional sequential imaging and the benefits of short acquisition times of spiral CT. Independently from the acquisition technique, iterative reconstruction (IR) techniques have the potential to increase image quality by reducing image noise. Thus, resulting in a smaller slice thickness as well as beam hardening artifacts [8, 9].

The aim of this study was to prospectively compare the diagnostic image quality between sequential cCT on a 4-slice MDCT and cCT performed in a spiral mode on a an 192-slice MDCT system in combination with a novel advanced model IR technique using intra-individually identical radiation dose levels.

## Material and Methods

This single-center study, consisting of a retrospective and a prospective part, was approved by the institutional review board (IRB) “Medizinische Ethikkommission II der Medizinischen Fakultät Mannheim” and complies both with the Declaration of Helsinki and the Health Insurance Portability and Accountability Act (HIPAA). Regarding the retrospective part of the study, the IRB did not require written informed consent, hence none was obtained. Nonetheless, in keeping with the IRB guidelines, exceeding care was taken for the relevant data to be anonymized and de-identified prior to the analysis. Regarding the prospective part of the study, the IRB required written informed consent. All of the obtained written consents were collected and filed in our database.

### Patient cohort

In this study, 35 patients (mean age 71.4 years ±13.1 years [range 30–90 years]; 19 male) with indications for clinical routine non-contrast enhanced cCT and a previous examination on a 4-slice Multi-Detector CT (MDCT) system within the last 12 month (retrospective part) were enrolled between December 2013 and August 2014 for cCT on a 3^rd^ generation Dual-Source-CT (DSCT) system (prospective part). Both inpatients and outpatients were included. Table 1 summarizes the indications for the cCT examinations.

**Table 1:** Patient demographics, clinical characteristics and image parameters.

**No. of patients**	35
**Mean age (SD), *y***	71.4 *(13*.*1)*
Range	30–90
**Sex, male:female**	1.2:1
**Indications for scanning, No. of patients**	
(Rule out) hemorrhage	17
Follow-up after surgery	11
(Rule out) hydrocephalus	8
Follow-up after cSDH	6
Stroke	5
Trauma	5
**CT acquisition**	
**MDCT**	
Data acquisition	sequential
Gantry tilting	yes
Scan direction	cranio-caudal
Detector collimation, *mm*	4 x 1
Rotation time, *sec*	3 x 1
Mean CTDIvol, *mGy*	64.67
Mean DLP, mGy*cm	961.62
Pitch factor	-
**DSCT**	
Data acquisition	spiral
Gantry tilting	no
Scan direction	cranio-caudal
Detector collimation, *mm*	2 x 96 x 0.6
Rotation time, *sec*	1
Mean CTDIvol, *mGy*	64.6
Mean DLP, mGy*cm	1076.0
Pitch factor	0.55

CTDI: volume computed tomography dose index; DLP: dose-length product

Note: Some patients had multiple indications for cCT

### CT acquisition and image reconstruction

The volume CT dose index (CTDI_vol_) and dose-length-product (DLP) were recorded for every CT examination (Table 1). The initial clinical routine cCT examinations within the 12 previous months were all performed on a 4-slice MDCT system (SOMATOM Volume Zoom, Siemens Healthcare Sector, Forchheim, Germany) using a standard sequential technique with the following scan parameters: 120 kV tube voltage; 270 mAs tube current time product; 4 x 1 mm detector collimation; 3 x 1 sec rotation time, cranio-caudal scan direction. CT raw data was reconstructed with a slice thickness of 4 mm using filtered-back projection and a dedicated brain tissue convolution kernel (H40s medium).

The spiral cCT acquisitions of all patients were performed on a 3^rd^ generation DSCT system (Somatom FORCE, Siemens Healthcare Sector, Forchheim, Germany) using the following scan parameter: 120 kV tube voltage, 398 mAs tube current time product, 2 x 96 x x 0.6 mm detector collimation, 1 sec rotation time, pitch factor 0.55; cranio-caudal scan direction. To guarantee for the exact same intra-individual radiation dose the tube current was individually adapted according the CTDI_vol_ that was applied during the initial sequential cCT until the same CTDI_vol_ was reached. Since the DSCT system does not allow for tilting the gantry the patients were positioned into a dedicated head cup in order to exclude the orbita from the scan field. The CT raw data of the spiral DSCT examinations was reconstructed with a slice thickness of 4 mm using FBP with the corresponding brain tissue convolution kernel “Hr38”. Further, advanced model iterative reconstruction [(ADMIRE) Siemens Healthcare Sector, Forchheim, Germany] with strength levels 1–5 were employed.

### Assessment of objective and subjective image quality

All cCT datasets were transferred to an image viewing workstation (Aycan Osirix Pro [aycan Digitalsysteme GmbH, Wuerzburg, Germany]). Objective image quality was assessed in two predefined regions by placing identical regions of interest (ROIs)–white matter (WM) and caudate nucleus (NC), excluding pathology that could affect results (e.g. foreign bodies, blood products).

Within the ROIs one radiologist (_._) measured image noise, defined as the standard deviation of the measured Hounsfield units (HU), and the mean attenuation (signal) I HU.

The signal-to-noise (SNR) ratio was calculated using these measurements. Subjective image quality was independently rated by two experienced neuroradiologists (__;__), working independently and blinded to the reconstruction method. Evaluation parameters/criteria were as follows: grey/white matter differentiation, delineation of anterior/posterior part of internal capsule on both sides, evaluation of subjective image noise and artifacts, delineation of ventricular system, subarachnoid space, brainstem, cerebellar hemisphere and brain lesions (if present). The subjective image criteria and 4-point-Likert-scale used are summarized in Table 2. The best of all five IR images of spiral DSCT was identified and compared to sequential MDCT images.

**Table 2:** 4-grade scoring system of the subjective evaluation parameters.

Structure	Score 1	Score 2	Score 3	Score 4
**Gray/white matter**	Perfect differentiation	Very good differentiation	Delineation not perfect but	Differences just depictable
**differentiation**			acceptable for diagnostic purboses	
**Anterior/posterior part**	Perfect delineation	Very good visualization,	Unsharp borders but	Visualization just possible
**of internal capsule**	well-defined anatomy	well-defined anatomy	different structures already visible	
**Subjective image noise**	Little to no noise	Optimum noise	Noisy, but permits evaluation	Noisy, degrades image so that
				no evaluation possible
**Ventricular system**	Perfect delineation	Very good visualization,	Unsharp borders but	Visualization just possible
	well-defined anatomy	well-defined anatomy	different structures already visible	
**Subarachnoid space**	Perfect delineation	Very good visualization,	Unsharp borders but	Visualization just possible
	well-defined anatomy	well-defined anatomy	different structures already visible	
**Infra- and supratentorial artifacts**	Free of visible artifacts	Some artifacts but quality	Substantial decrease in	Image beeing totally
		not substantially impaired	image quality	impaired by artifacts
**Cerebellar hemisphere**	Perfect delineation	Very good visualization,	Unsharp borders but	Visualization just possible
	well-defined anatomy	well-defined anatomy	different structures already visible	
**Brainstem**	Perfectly visible structure	Good but not perfect	Visible but not in detail	No anatomic detail
**Brain lesions (e.g. Lacunar infarct)**	Perfectly visible structure	Good but not perfect	Visible but not in detail	No anatomic detail

### Statistical analysis

Statistical analyses were performed using SPSS 20.0 (SPSS Inc., Chicago, IL). Normal distribution of the datasets was tested using Shapiro–Wilk test and Q-Q plots. If otherwise not indicated normally distributed data are presented as mean ± standard deviation (SD). Non parametrical data (e.g. image quality scores) are presented as median with 25^th^ -75^th^ percentile interquartile range (IQR) and were compared using the Wilcoxon sign-rank analysis. P-values <0.05 were considered statistically significant. In case of multiple comparisons the significance threshold was adjusted using Bonferroni-correction. IR method with the highest mean SNR and best subjective image quality was chosen and compared to spiral DSCT images by Wilcoxon sign-rank analysis.

## Results

All 70 studies were successfully conducted and demonstrated diagnostic image quality at 4 mm. Patient demographics and clinical characteristics, and image parameters are summarized in Table 1.

### Quantitative image quality

**Fig 1:**
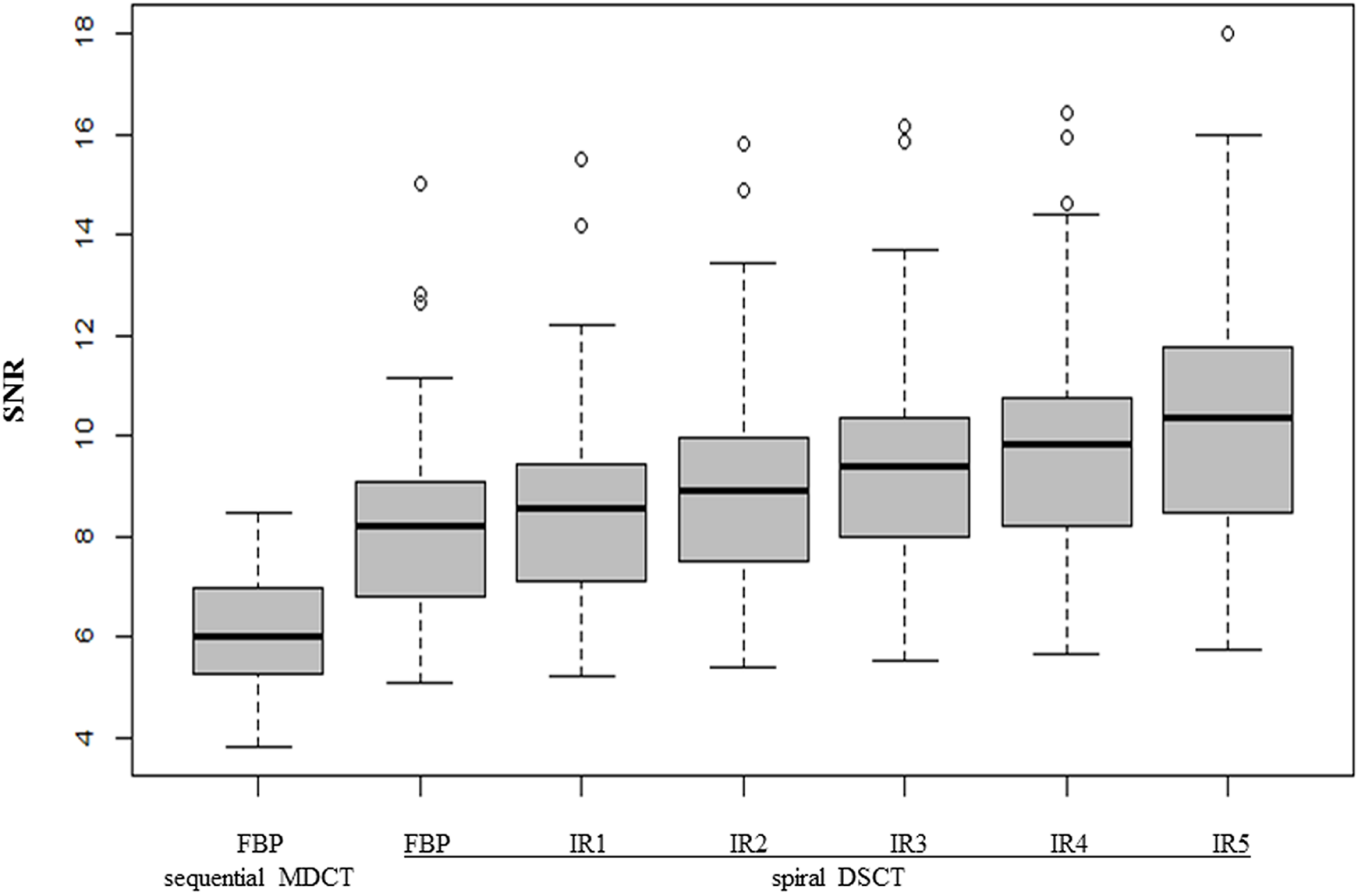
SNR of FBP and all levels of iterative reconstruction of spiral cCT versus sequential cCT in white matter (WM): There was a highly significant improvement of SNR (Wilcoxon sign-rank test, p*_Bonferroni_<0.05).

Comparison between the sequential cCT and all spiral DSCT reconstructions showed a significantly increased SNR in the WM and the NC (Wilcoxon sign-rank test, p**_Bonferroni_ <0.01). On the sequential cCT reconstructed with FBP mean SNR in the white matter was 6.22 (±1.12). On spiral cCT, median SNR of white matter reconstructed with FBP was 8.21 (IQR: 9.21–6.71 = 2.50). The median SNR in the WM (S1 File) increased in the course of higher strength levels of IR; SNR IR 1: 8.54 (IQR: 9.74–7.00 = 2.74), SNR IR 2: 8.93 (IQR: 10.13–7.48 = 2.65), SNR IR 3: 9.42 (IQR: 10.58–7.99 = 2.59), SNR IR 4: 9.85 (IQR: 10.76–8.20 = 2.56), SNR IR 5 10.34 (IQR: 11.94–8.34 = 3.53) (Fig 1).

The pairwise comparison using Bonferroni correction showed a statistically significant difference particularly between higher and lower strength levels of iterative reconstruction as well as between spiral and sequential cCT (Wilcoxon sign-rank test, p*_Bonferroni_<0.05) (for details, see Table 3A).

**Table 3:** Pairwise comparison of SNR in sequential and spiral cCT.

		sequential			spiral			
		**FBP**	**FBP**	**IR 1**	**IR 2**	**IR 3**	**IR 4**	**IR 5**
sequential	**FBP**	**1,0**	0,00001109	0,00000040	0,00000040	0,00000040	0,00000040	0,00000040
	**FBP**	0,00001109	**1,0**	0,00001109	0,00000221	0,00000006	0,00000040	0,00000006
	**IR 1**	0,00000040	0,00001109	**1,0**	0,00000006	0,00000001	0,00684086	0,00684086
spiral	**IR 2**	0,00000040	0,00000221	0,00000006	**1,0**	0,00000040	0,00000006	0,00000221
	**IR 3**	0,00000040	0,00000006	0,00000001	0,00000040	**1,0**	0,00234579	0,00000221
	**IR 4**	0,00000040	0,00000040	0,00684086	0,00000006	0,00234579	**1,0**	0,00001109
	**IR 5**	0,00000040	0,00000006	0,00684086	0,00000221	0,00000221	0,00001109	**1,0**
Pairwise comparison of SNR in caudate nucleus in sequential and spiral cCT								
		sequential			spiral			
		**FBP**	**FBP**	**IR 1**	**IR 2**	**IR 3**	**IR 4**	**IR 5**
sequential	**FBP**	**1,0**	0,00000221	0,00000221	0,00000221	0,00000040	0,00000001	0,00000006
	**FBP**	0,00000221	**1,0**	0,00000221	0,00000001	0,00000001	0,00000040	0,00000040
	**IR 1**	0,00000221	0,00000221	**1,0**	0,00000221	0,00000001	0,00000006	0,00000006
spiral	**IR 2**	0,00000221	0,00000001	0,00000221	**1,0**	0,00000221	0,00004976	0,00000040
	**IR 3**	0,00000040	0,00000001	0,00000001	0,00000221	**1,0**	0,00000221	0,00000221
	**IR 4**	0,00000001	0,00000040	0,00000006	0,00004976	0,00000221	**1,0**	0,00001109
	**IR 5**	0,00000006	0,00000040	0,00000006	0,00000040	0,00000221	0,00001109	**1,0**

Note: Difference of signal to noise ratio is significant at the 0.05 level adjusted for multiple comparisons using Bonferroni-correction

**Fig 2:**
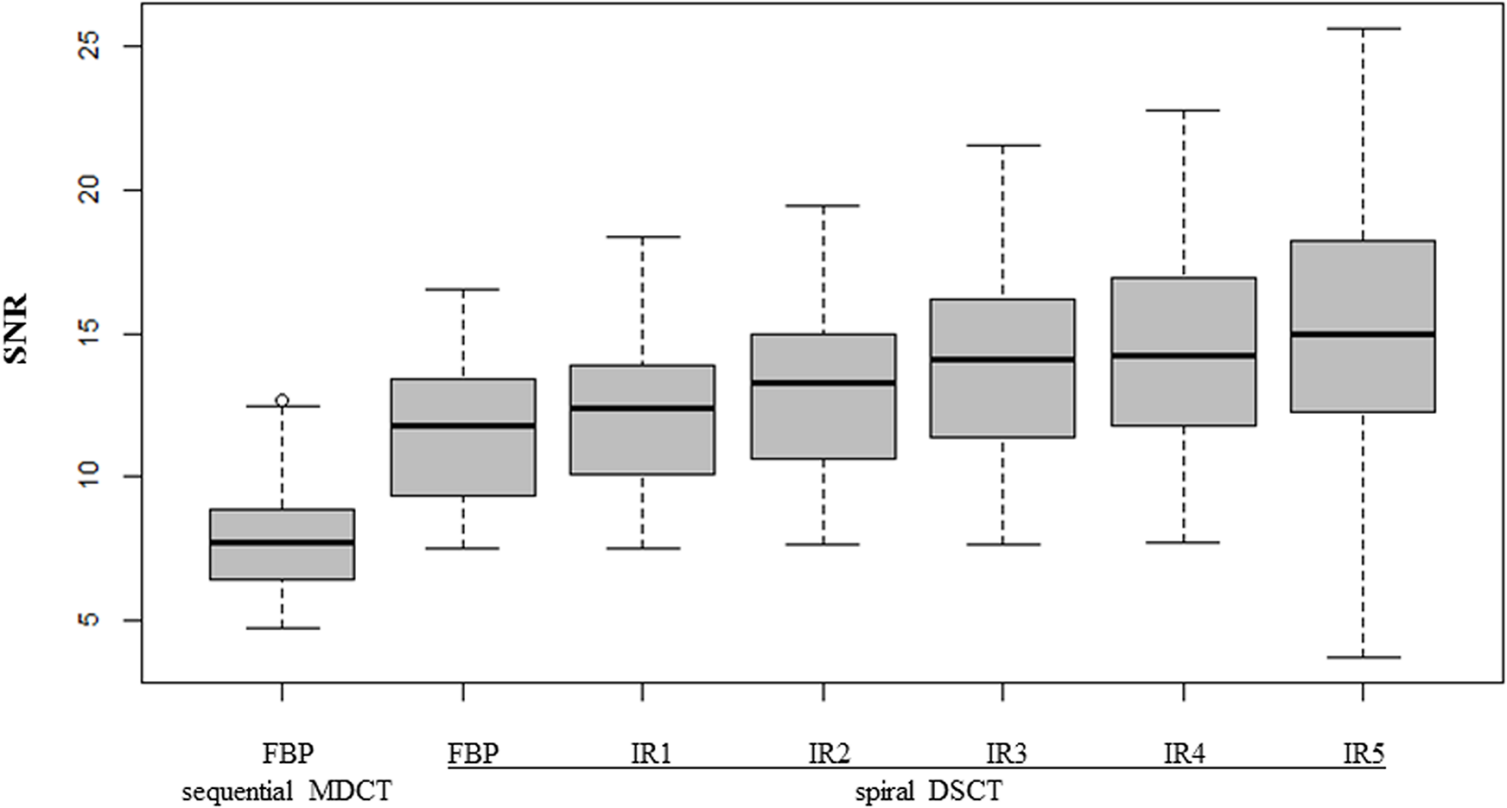
SNR of FBP and all levels of iterative reconstruction of spiral cCT versus sequential cCT in caudat nucleus (NC): There was a highly significant improvement of SNR (Wilcoxon sign-rank test, p*_Bonferroni_ <0.05).

The mean SNR in the NC on sequential cCT reconstructed with FBP was 7.89 (±2.0) (S2 File). Similar to the above-mentioned findings, image noise in the NC decreased and SNR increased with higher strength levels of iterative reconstruction on DSCT. SNR of FBP: median 11.78 (IQR: 13.49–9.29 = 4.20), mean SNR IR 1: 12.40 (±2.75), mean SNR IR 2: 13.04 (±2.93), mean IR 3: 13.92 (±3.23), mean SNR IR 4: 14.47 (±3.39), mean SNR IR 5: 15.48 (±4.31) (Fig 2).

The consecutive pairwise comparison using Bonferroni correction showed a statistically significant difference particularly between higher and lower levels of strength levels of iterative reconstruction as well as between spiral and sequential cCT (Wilcoxon sign-rank test, p*_Bonferroni_ <0.05) (for details, see Table 3B).

In comparison to the 4-slice MDCT, FBP in combination with iterative reconstruction on a 3^rd^ generation DSCT system shows an overall (WM and NC) mean improvement of SNR ratios of 61.65%. Increasing objective image quality is related to higher strength levels of iterative reconstruction; mean improvement (WM and NC) of DSCT towards MDCT is 42.09% in FBP, 49.43% in IR 1, 56.59% in IR 2, 65.92% in IR 3, 72.69% in IR 4 and 83.16% in IR 5.

### Qualitative image quality assessment

Table 4 shows the mean values of the averaged qualitative ratings per criteria in the nine rated aspects within one reconstruction level reported separately for the two observers (S3 File).

**Table 4:** Mean values of ratings averaged over all examined regions reported separately for the two observers.

Structure	*Mean* score (radiologist I)						Sequential MDCT	*Mean* (radiologist II)						Sequential MDCT
	Spiral DSCT							Spiral DSCT						
	FBP	IR 1	IR 2	IR 3	IR 4	IR 5		FBP	IR 1	IR 2	IR 3	IR 4	IR 5	
**Gray/white matter differentiation**	3,06	2,80	2,14	2,03	1,09	1,03	3,46	2,94	2,60	2,06	2,00	1,60	1,09	2,97
**Anterior/posterior part of IC**	3,09	2,74	2,20	2,11	1,26	1,06	3,31	2,94	2,91	2,09	2,03	1,34	1,20	2,97
**Subjective image noise**	3,06	2,71	2,29	2,06	1,20	1,06	3,40	2,94	2,17	2,03	2,00	1,06	1,06	2,97
**Ventricular system**	3,00	2,71	2,31	2,03	1,26	1,06	2,97	2,94	2,26	2,03	1,97	1,17	1,06	2,91
**Subarachnoid space**	2,94	2,54	2,23	2,06	1,20	1,03	2,97	3,00	2,17	2,03	1,91	1,06	1,00	2,97
**Infra- and supratentorial artifacts**	2,60	2,57	2,54	2,49	2,37	2,34	2,77	3,00	2,97	2,94	2,94	2,83	2,83	3,26
**Cerebellar hemisphere**	2,97	2,43	2,26	2,23	1,69	1,43	2,97	3,00	2,11	2,06	2,06	1,37	1,17	3,00
**Brainstem**	2,97	2,94	2,57	2,14	1,86	1,66	3,00	3,00	2,91	2,14	2,03	1,89	1,89	3,00
**Brain lesions (e.g. lacunar infarct)**	2,97	2,62	2,29	2,06	1,32	1,24	2,97	2,97	2,18	2,06	1,97	1,32	1,09	3,06
**Cummultive Mean Score**	2,96	2,67	2,32	2,13	1,47	1,32	3,09	2,97	2,48	2,16	2,10	1,52	1,37	3,01

Note: a smaller score (1–4) represents a better subjective image quality.

**Fig 3:**
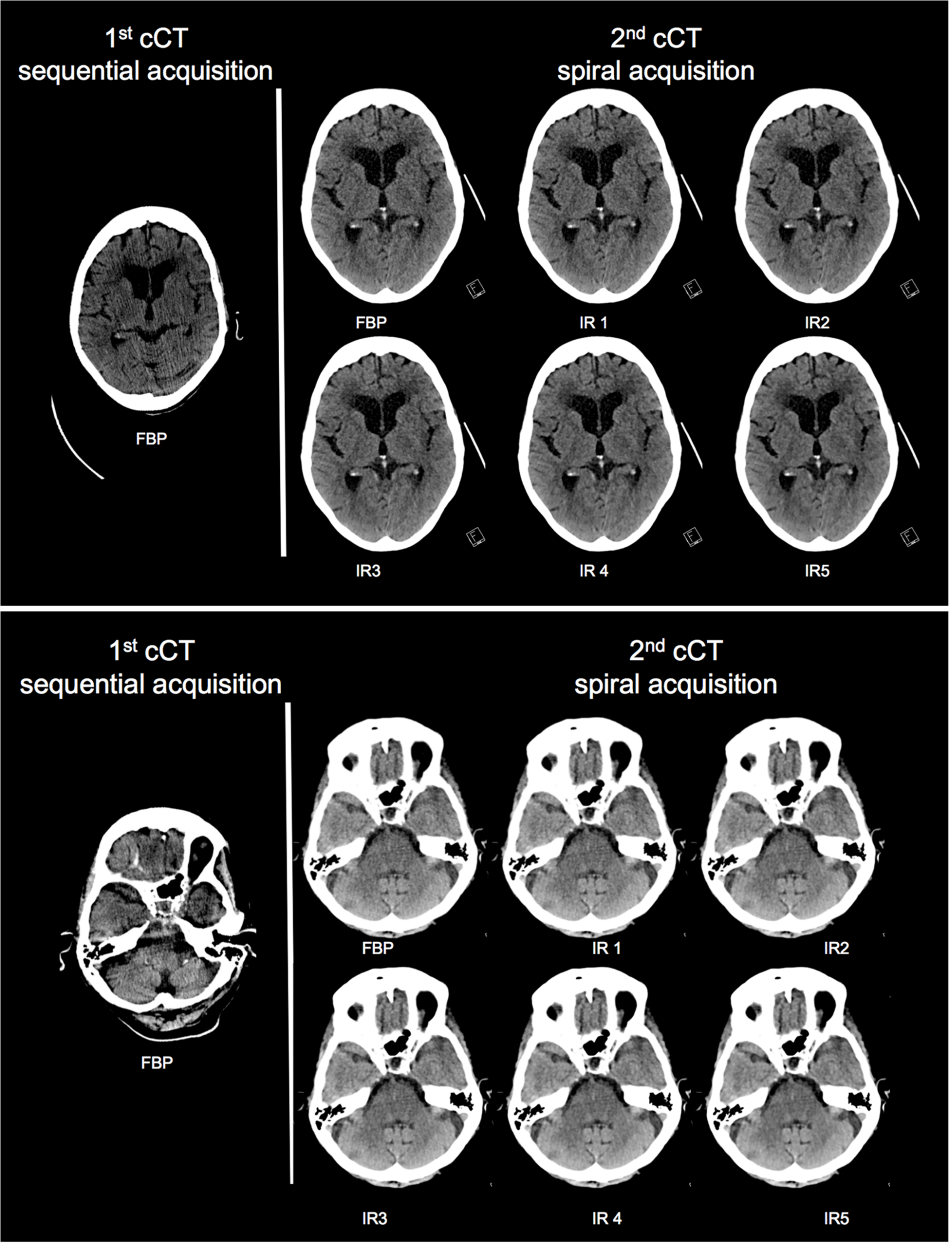
Direct intra-individual comparison of standard 4-slice MDCT and 3rd generation DSCT cCT. *Left*: 94 year old patient with a cCT on a 4 slice MDCT system in a sequential acquisition mode. *Right*: 2 x 192 slice DSCT cCT of the same patient examined at the same day later due to neurological deterioration using the identical CTDI_vol_ and iterative image reconstruction with strength level 1–5.

Mean grading continuously increased within different iterative strength levels by both readers. In every assessment criteria, the sequential CT had the worst rating (i.e.: highest score). Moreover, there was a trend to positive correlation that both readers allocated smaller scores (i.e. better readability) for higher strength levels of IR (FBP>IR 1>IR 2>IR 3>IR 4>IR 5). As a result, IR 5 was rated best by both readers, as evident in all structures. Consequently IR 5 technique was chosen based on its highest WM- and NC-mean SNR, as well as best subjective image quality profile for pairwise comparison with the spiral CT (regarding the scoring aspects). IR 5 method had a statistically highly significant better subjective image quality in all examined scoring category than the sequential CT (Wilcoxon sign-rank analysis—for details see Table 5). Fig 3 shows the direct intra-individual comparison of standard 4-slice MDCT and 3^rd^ generation DSCT cCT; clearly, the quality of the image testifies to the exceptional resolution of the new spiral CT as well as the improvements of the IR technique.

**Table 5:** Comparison of spiral iterative reconstruction strength level 5 (IR5) to sequential MDCT.

Structure			*Mean* score (radiologist I)			*Mean* (radiologist II)		
			IR 5	Sequential MDCT	p	IR 5	Sequential MDCT	p
**Gray/white matter differentiation**			1,03	3,46	0,00000011	1,09	2,97	0,00000004
**Anterior/posterior part of IC**			1,06	3,31	0,00000007	1,20	2,97	0,00000010
**Subjective image noise**			1,06	3,40	0,00000010	1,06	2,97	0,00000002
**Ventricular system**			1,06	2,97	0,00000004	1,06	2,91	0,00000004
**Subarachnoid space**			1,03	2,97	0,00000003	1,00	2,97	0,00000001
**Infra- and supratentorial artifacts**			2,34	2,77	0,00129690	2,83	3,26	0,00062902
**Cerebellar hemisphere**			1,43	2,97	0,00000010	1,17	3,00	0,00000003
**Brainstem**			1,66	3,00	0,00000008	1,89	3,00	0,00000002
**Brain lesions (e.g. lacunar infarct)**			1,24	2,97	0,00000008	1,09	3,06	0,00000006
**Cummultive Mean Score**			1,32	3,09		1,37	3,01	

Note: IR series 5 with the highest mean SNR both in gray- and white matter as well with the best subjective image quality was chosen and compared to spiral DSCT images by Wilcoxon sign-rank analysis. Differences are given for both readers.

### Dose measurements

The mean CTDI_vol_ on the sequential MDCT system was 64.67 mGy; while the mean CTDI_vol_ on the spiral DSCT system was 64.6 mGy. The mean DLP on MDCT was 961.62 mGy*cm, compared to a DLP on the DSCT of 1076.0 mGy*cm.

## Discussion

Although computer power has only recently evolved to allow implementation of IR in clinical routine, the concept of IR algorithms were used for the first time almost four decades ago [10]. Previous studies on IR showed, that new models of reconstruction significantly improved diagnostic accuracy and reduced image noise and radiation dose [9, 11–21]

In this study we aimed to prospectively compare objective and subjective image quality of intra-individual sequential and spiral cCT, using traditional FBP and advanced modeled iterative reconstruction technique in comparison with the standard 4-slice MDCT system. cCT imaging of high quality and resolution of structures containing low contrast differences requires sufficiently high radiation doses in order to avoid statistical noise which obscures differentiability of adjacent structures [6]. Therefore, it is crucial to compare sequential and spiral acquisitions cCT in studies performed with equal dose. Our results clearly indicate that in comparison with standard sequential cCT acquisition at identical dose levels, spiral cCT acquisitions on a state-of-the-art CT system in combination with IR results in increased objective as well as subjective image quality. Additional weight to this conclusion is added by the advantageous use of intra-individual comparison of both modalities.

In the past, several objections against spiral cCT were put forward which led to a low acceptance thereof especially regarding routine examinations in the field of neuroradiology [5, 6]. However, these objections are successfully combated using 3^rd^ generation DSCT in combination with current IR, as the following discussion shows: Firstly, Bahner *et al*. [5] objected that the delineation of structures with low contrast differences (e.g. grey and white matter) might be inadequate. Likewise, van Straten and colleagues [22] could not show that spiral CT improves differentiation of gray/white matter, nor visualizing hypodense lesions. However, they could show a positive trend towards spiral CT regarding artifacts and subjective “overall image quality”. In contrast to both investigators, Bahner and van Straten *et al*., our investigation showed an improvement when using spiral CT in almost all subjective criteria like gray/white matter differentiation, delineation of anatomical structures, subarachnoid spaces and hypodense lesions. Statistically, the subjective image quality rating of the 3^rd^ generation DSCT was significantly higher when compared to the 4-slice MDCT system (all p<0.05). Among the subjective ratings of all IR levels, the images with an IR strength level of 5 and 4 achieved significantly higher subjective image quality scores when compared to lower strengths (1–3) as well as FBP images. Objective means of comparison of both spiral and conventional CT were carried out by investigating the commonly used signal-to-noise ratio (SNR). The mean SNR of the 3^rd^ generation DSCT system was statistically significantly higher when compared to the 4-slice MDCT system using the same CTDI_vol_ with an overall (across white and grey matter) mean improvement of 61.65%, regarding FBP (MDCT) and all levels of IR (DSCT); but an even higher improvement of 83.16% only regarding IR 5. However, it is to note that IR 5 with the best SNR both in WM and NC as well as qualitative image evaluation had the highest variance both for WM and NC (see Figs 1 and 2) among all techniques. Again, this further adds to the validity of using the new generation of spiral CTs, contrary to past investigations [5].

Secondly, studies in the past encountered artifacts localized close to the skull in spiral cCT [5, 23, 24]. These artifacts could easily be mistaken for subarachnoidal bleeding or acute hematoma. The physics of linear interpolation which are required for spiral cCT data processing are likely to be the source of these artifacts [25]. This, of course, has crucial clinical implications, as it presents a pressing dilemma. Patients presenting with a trauma in the clinic would notably benefit from decreased scanning times associated with spiral cCT. However, these patients are also the most likely to present with symptoms that are resembling subarachnoidal bleeding or acute hematoma. Therefore, using a spiral cCT of the older generation appeared to be rather irresponsible, due to the increased likelihood of a false-positive diagnosis. However, when using the 3^rd^ generation DSCT and current IR, the described artifacts are significantly less present. Consequently, the spiral cCT using the new DSCT and current IR technique has major implications for patients presenting with trauma, as it can further contribute to reducing the time from injury, to diagnosis and finally to critical intervention or therapeutical management of the disease. The integration of radiologists in ‘trauma teams’ testifies to this effort [26]. Of greater therapeutic impact is the utilization of new generation spiral cCT in patients presenting with a suspected acute ischemia. In order to appropriately treat the patient it is imperative to differentiate the stage of suspected ischemia as well as discriminate whether a hemorrhage might be present [27, 28]. This in turn, necessitates the usage of an imaging procedure that has the highest accuracy and or produces only neglectable artefacts. Our results suggest that the new 3^rd^ generation DSCT in combination with the highest iterative techniques meets these requirements.

Thirdly, Kuntz *et al*. have argued that there is no real demand for rapid scanning as brain and spine imaging lacks of motion artifacts due to respiration [6]. This, however, is only true for some cases. As described above, patients presenting with trauma require immediate diagnosis in order to reduce time from injury to intervention [26]. Moreover, rapid scanning is beneficial for patients that are difficult to handle due to agitation, which include patients with acute neurological disorders such as intracranial hemorrhage or cerebral ischemia.

Our study has some limitations that should be considered. First, the investigated patient cohort was of moderate size. Second, we only analyzed objective and subjective image quality within spiral and sequential CT rather than diagnostic accuracy. Prospective studies with greater patient cohorts are needed to further elucidate the consequences of IR on patient outcomes and diagnostic accuracy. Third, there was no IR available in spiral acquisition mode. Finally, previous studies have described the subjective image quality as unfamiliar or often characterized these as “plastic” or “waxy” when using IR techniques [9, 29]. While we did not directly investigate this specific parameter, we nonetheless addressed this phenomenon indirectly by our subjective evaluation. As our results suggest, this phenomenon does not constitute as significant detrimental factor, which is likely to be compensated by the reduced noise, among other improvements.

## Conclusions

In conclusion, spiral cCT in combination with state-of-the-art iterative reconstruction techniques has significant advantages over sequential CT techniques and therefore is likely to pave the way for the implementation of spiral CTs in cranial neuroradiology as a standard procedure.

## Supporting Information

S1 File Mean signal-to-noise ratios in the white matter (WM). Column A: patient IDs; Column B: Mean SNR in the WM on the 4-slice Multi-Detector CT; Column C-H: Mean SNR in the WM on Dual-Source-CT with different levels of reconstruction (FBP = filtered backward projection; IR 1–5 = iterative reconstruction level 1–5).
(XLSX)

S2 File Mean signal-to-noise (SNR) ratios in the caudate nucleus (NC). Column A: patient IDs; Column B: Mean SNR in the NC on the 4-slice Multi-Detector CT; Column C-H: Mean SNR in the NC on Dual-Source-CT with different levels of reconstruction (FBP = filtered backward projection; IR 1–5 = iterative reconstruction level 1–5).
(XLSX)

S3 File Qualitative image quality assessment. Column A: patient IDs, column B-DW: qualitative ratings per criteria observers (MR = Gray/white matter differentiation; BasGgl = Anterior/posterior part of IC; Rau = Subjective image noise; Vent = Ventricular system; SAR = Subarachnoid space; Art = Infra- and supratentorial artifacts; Cerbll = Cerebellar hemisphere; BS = Brainstem; Laes = Brain lesions) in the nine rated aspects within one reconstruction level (DSCT: FBP = filtered backward projection; AM 1–9 = iterative reconstruction level 1–5; MDCT: VZ) reported separately for the two observers (W = observer 1; F = observer 2).
(XLS)
